# Nonalcoholic Steatohepatitis: Involvement of the Telomerase and Proinflammatory Mediators

**DOI:** 10.1155/2015/850246

**Published:** 2015-07-26

**Authors:** Rim Serhal, George Hilal, George Boutros, Joseph Sidaoui, Layal Wardi, Salah Ezzeddine, Nada Alaaeddine

**Affiliations:** ^1^Faculty of Medicine, Saint Joseph University, Regenerative Medicine and Inflammation, Beirut 11-5076, Lebanon; ^2^Faculty of Medicine, Saint Joseph University, Metabolism and Oncology Lab, Beirut 11-5076, Lebanon; ^3^Gastroenterology Unit, Military Hospital, Ghoubeiry 2, Beirut 25-413, Lebanon

## Abstract

Nonalcoholic steatohepatitis or NASH is an excessive accumulation of fat in hepatocytes accompanied by inflammation and hepatic injury. Proinflammatory molecules such as IL-17, CCL20, S100A8, S100A9, and S100A8/A9 have been shown to be implicated in many types of cancer. Telomerase activity has been found to be associated with chronic inflammation and cancer. NASH can progress to fibrosis then cirrhosis and finally to hepatocellular carcinoma (HCC). Our objective is to try to find a relation between inflammation and the progression of NASH into HCC. We found that there was a significant elevation in the telomerase activity, detected by real-time PCR, between NASH and fibrotic NASH in the liver biopsies of patients. The expression of S100A8, S100A9, S100A8/A9, CCL20, and IL-17, detected by ELISA, is significantly increased in NASH patients with fibrosis in comparison with controls. But, in NASH patients, S100A9, S100A8/A9, and IL-17 only are significantly elevated in comparison with controls. The same, on the mRNA level, expression of IL-17, detected by RT-PCR, is significantly elevated in NASH patients in comparison with controls. Therefore, there is a direct link between the expression of IL-17, CCL20, telomerase, S100A8, and S100A9 in the fibrotic condition and the progression towards cancer.

## 1. Introduction

Nonalcoholic steatohepatitis (NASH) is characterized by steatosis, hepatocellular injury, and parenchymal and portal inflammation [1]. Inflammation is thought to be the main factor in the development of NASH. Proinflammatory mediators such as IL-6 and TNF-*α* have been shown to be associated with increased severity of hepatic inflammation [2, 3]. Expression of IL-6 and TNF-*α* is elevated in NASH, liver cirrhosis, and hepatocellular carcinoma [4]. IL-17 is another proinflammatory cytokine associated with hepatic steatosis and proinflammatory response in nonalcoholic fatty liver disease (NAFLD) and has been suggested to facilitate the transition from simple steatosis to steatohepatitis [5]. Those inflammatory cytokines perpetuate inflammation and may contribute to the differentiation of NASH to fibrosis. However, the most worrying scenario is the potential for the progression of NASH to cirrhosis then hepatocellular carcinoma (HCC) [6].

HCC is the most common form of cancer accounting for 70% to 85% of the total liver cancer mortality [7]. The precise prevalence of HCC eminent from NASH is unknown. However, several studies have reported the occurrence of HCC from cirrhotic and noncirrhotic NASH patients [8]. A study from the US health care reported that 38.2% of patients with HCC originated from the complications of nonalcoholic fatty liver disease [9]. This high incidence constrains the need to understand the mechanism responsible for progression of NASH to HCC.

S100A8 and S100A9, members of the S100 proteins, are small calcium-binding proteins that are highly expressed in neutrophil and monocyte cytosol and are found to be overexpressed during inflammatory conditions [10, 11] and in some types of cancer such as pancreatic, colorectal, and gastric cancer [12–14]. The heterodimer formed by these two S100 proteins, S100A8/S100A9, is also implicated in inflammatory diseases and has been suggested as a biomarker for monitoring inflammation and disease activity [15]. Not only this, but also several experimental data have shown a link between the upregulation of S100A8, S100A9, and S100A8/S100A9 in inflammatory associated cancer [16].

Telomerase is a ribonucleoprotein complex that catalyzes the elongation of telomeres. This system counteracts telomere erosion physiologically and thus prevents cellular senescence. Most somatic cells have very low or undetectable telomerase activity. In contrast, cells with high proliferative potential are characterized by a high enzymatic activity, such as activated T cells, stem cells, and tumor cells [17–19]. Telomerase is an enzyme involved in cellular aging [20]; it plays a role in the transformation of cells from the precancerous stage to carcinoma [21]. Testing in vitro and in vivo has demonstrated that, without telomerase, cancer cannot develop [22]. Telomerase activity has been detected in a great percentage of solid tumors including hepatomas [23] and gastric and colon carcinomas [24–26] and it is suggested to be a tool for diagnosis and prediction of recurrence in HCC [27].

The progression of NASH to HCC is frequently reported [28–30]; what the factors involved are and how they contribute to this progression are not fully understood. Is the presence of cytokines such as interleukin-17 (IL-17) or S100 calgranulins known for their role in several inflammatory diseases sufficient to predict the evolution of NASH to cirrhosis and neoplastic transformation? Is the activation of telomerase in the liver of NASH patients necessary for impaired proliferation and malignant transformation?

The aim of this study was to evaluate the telomerase activity, calprotectin, and proinflammatory cytokines in NASH patients in order to try to find a correlation between inflammation in NASH and development to HCC.

## 2. Materials and Methods

### 2.1. Patients

Our study group was thirty-nine diagnosed patients with NASH attending the Military Hospital, Beirut, Lebanon. The diagnosis of NASH was based on the following criteria: (1) intake of less than 20 g of ethanol per day, (2) a positive ultrasonography for steatosis, (3) biopsy proven steatohepatitis, steatosis, inflammatory infiltrates, and ballooning degeneration with or without Mallory bodies or pericellular/perivenular fibrosis, and (4) appropriate exclusion of other liver diseases patients also had to have an increase in aspartate aminotransferase (AST) and alanine aminotransferase (ALT) of 1 to 4 times the upper limit of normal, with an AST/ALT ratio of <1. Patients with chronic inflammatory disease, active infection, recent surgery or trauma, or history of chronic drug use (nonsteroidal anti-inflammatory drugs, corticosteroids, high-dose estrogens, methotrexate, tetracycline, or amiodarone) were excluded. Thirty healthy, nonobese (BMI < 30 kg/m^2^) volunteers who were evaluated for the absence of liver disease by means of ultrasound and AST and ALT levels served as controls. The Institutional Review Board at each center approved the study protocol and written informed consent was obtained from each patient.

### 2.2. Laboratory Evaluation

The laboratory evaluation in all patients included a blood cell count and measurement of total cholesterol, LDL, HDL, aspartate aminotransferase (AST), alanine aminotransferase (ALT), C reactive protein (CRP), total cholesterol, and total bilirubin, glucose, and insulin.

### 2.3. Telomerase Activity Measurement

Telomerase activity was performed on liver biopsy and was measured by real-time PCR using a Quantitative Telomerase Detection Kit (allied Biotech, Inc), which is based on the ability of telomerase presented in cell extracts to synthesize telomeric repeats onto an oligonucleotide substrate, and the resultant extended product is subsequently amplified by polymerase chain reaction (PCR). Generated PCR products are then visualized using highly sensitive DNA fluorochromes Sybr Green. Detection of PCR products is measured following the binding of Sybr Green dye to double-strand DNA.

Liver biopsies extracts were prepared according to the manufacturer protocol. Briefly, liver biopsies were washed twice with cold PBS; then, they were lysed with an appropriate volume from the provided lysis buffer. After 30 minutes of incubation on ice, the suspension was centrifuged for 30 min at 4°C at 12000 ×g. The supernatant was then aliquoted for further telomerase activity and protein determination.

### 2.4. Measurement of S100 A8, S100A9, and S100A8/9

The serum levels of S100A8, S100A9, and S100A8/9 were measured in duplicate by the quantitative sandwich enzyme linked immunoassay commercially available by BMA Biomedicals (Rheinstrasse, Switzerland).

### 2.5. Measurement of IL-17 and CCL20

The levels of IL-17, CCL20, and TNF-*α* were measured in the serum of patients and healthy control by a commercial high sensitivity enzyme-linked immunosorbent assays (ELISA) (R&D, Abingdon, United Kingdom), according to the manufacturer protocol without modification. The sensitivity of the IL-17 and CCL-20 assays, as stated in the instructions of the manufacturer, was 7.8 pg/mL and 15 pg/mL, respectively.

### 2.6. RNA Extraction and Polymerase Chain Reaction

Total RNA from liver biopsy was extracted using QIAamp RNA extraction kit (Qiagen Inc., Valencia, CA, USA). Complementary DNA (cDNA) was synthesized from 0.1 to 2.5 *μ*g of total RNA in a 20 *μ*L reaction using Omniscript Reverse Transcription Kit (Qiagen Inc., Valencia, CA, USA). The expression of IL-17 and GAPDH was assessed using polymerase chain reaction (PCR) technique and specific primers. However, 0.4 *μ*M from cDNA was added to 25 *μ*L of PCR master mix (Fermentas) containing 5 U/*μ*L of Taq polymerase, 4 mM of MgCl_2_ and 0.4 mM from deoxynucleotides, and 2 *μ*L of primers and the reaction total volume of 50 *μ*L was completed using nuclease-free water. The IL-17 primers were as follows: forward 5′-CAATGACCTGGAAATACCCAA-3′, reverse 5′-TGAAGGCATGTGAAATCGAGA-3′; the GAPDH primers (glyceraldehyde 3-phosphate dehydrogenase), forward 5′-TGGTATCGTGGAAGGACTCATGAC-3′, reverse 5′-ATGCCAGTGAGCTTCCCGTTCAGC-3′, as the internal control, were also carried out by PCR. PCR amplification was performed using ABI thermocycler and the reactions were subjected to 35 PCR cycles of 94°C for 30 seconds, 60°C for 30 seconds, and 70°C for 30 seconds followed by 7 minutes' extension step at 72°C. The PCR products were separated on 2% agarose gel electrophoresis and visualized by ethidium bromide staining using UVP BioDoc system (UVP: ultraviolet products).

### 2.7. Statistical Analysis

Statistical analysis was performed by *t*-test using the online statistical software Graph-Pad Quickcalcs (http://www.graphpad.com/quickcalcs/ttest1.cfm). Results are presented as means ± SEM for the number of patients indicated. Statistical relation between variables was done using univariate linear regression. HOMA-IR was calculated using the following formula: insulin × glucose/22, 5 [32]. The histological grading was scored from 0 to 3 ensuing the criteria implemented by Brunt et al., with the modification being introduced by Merat et al. [31, 33]. Four major histological features of steatohepatitis were noted: steatosis, hepatocyte ballooning, lobular inflammation, and portal inflammation. A NASH activity index (NAI) was calculated by summing the scores for these features, producing a number between 0 and 12 [31]. The score of fibrosis (0 to 4) was also calculated separately according to Brunt et al. [33]. The scoring of histological features is thorough in Table 2.

## 3. Results

### 3.1. Comparison of Clinical Parameters between the NASH and Healthy Volunteers

Table 1 shows the comparison of the parameters analyzed between the NASH patients and healthy volunteers. There was no difference in age and gender between patients. In laboratory evaluation, AST, ALT, CRP, and insulin were found to be significantly elevated in the NASH patients compared to controls. There was no significant difference in other laboratory data between the two groups.

### 3.2. Telomerase Activity in the Liver Biopsies of NASH and NASH Patients with Fibrosis

**Figure 1:**
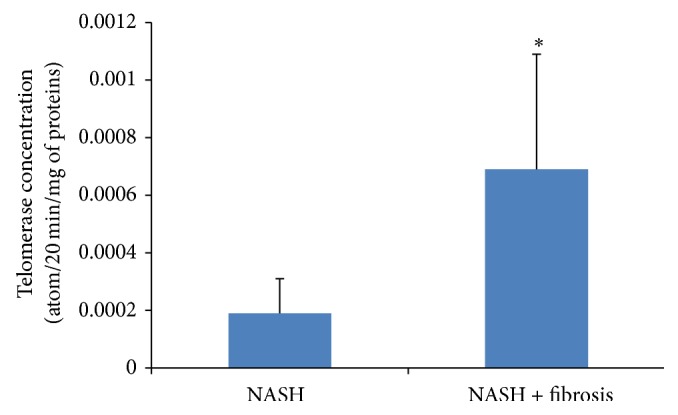
Detection of telomerase activity in liver biopsy extracts from NASH and NASH patients with fibrosis. Significant elevation in the biopsy of NASH patients with fibrosis in comparison with NASH patients with *P* value = 0.045 (^*^ *P* < 0.05).

Trying to find a way to detect early progression to HCC, we investigated the telomerase activity in the liver biopsies of NASH patients. Telomerase activity was detected in liver biopsies of NASH patients with fibrosis and was significantly increased (*P* = 0.045) in comparison to the level detected in the liver biopsies of patients with NASH without fibrosis (Figure 1).

### 3.3. Serum Level of Calgranulin A (S100A8), Calgranulin B (S100A9), and Calprotectin S100A8/S100A9 in NASH Patients

**Figure 2:**
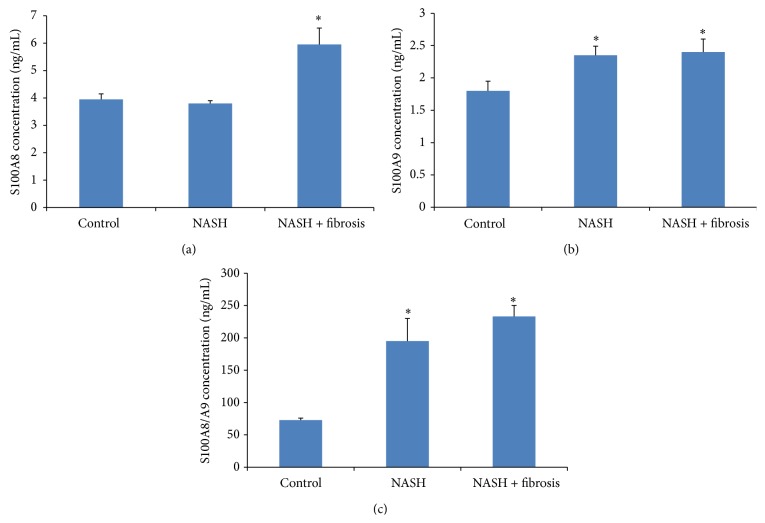
Levels of S100A8, S100A9, and S100A9/A9 in the serum of NASH and NASH patients with fibrosis in comparison with healthy controls. S100A8 was measured by ELISA as described in Section 2. Data represent mean ± SEM in triplicate (ELISA) from 39 different donors' serum from NASH patients and healthy volunteers. (a) S100A8 in NASH patients with fibrosis was significantly increased. Unpaired *t*-test; *P* = 0.008. (b) S100A9 in NASH and NASH patients with fibrosis was significantly increased. Unpaired *t*-test; *P* = 0.0056 and *P* = 0.0168, respectively. (c) S100A8/A9 in NASH and NASH patients with fibrosis was significantly increased. Unpaired *t*-test; *P* = 0.0001 and *P* = 0.0001, respectively (^*^ *P* < 0.05).

Since S100A8 and S100A9 are coexpressed in human HCC [34], we sought investigating the expression of S100A8, S100A9, and their complex in the serum of NASH patients and NASH patients with fibrosis. Indeed, there was no increase in S100A8 level in the serum of patients with NASH compared to control; however, the levels of S100A9 and S100A8/A9 were significantly elevated in patients with NASH with a *P* value < 0.01 (*P* = 0.0168 and *P* = 0.0001, resp.). But, in NASH patients with fibrosis, the level of S100A8, S100A9, and S100A8/A9 is significantly increased in comparison with controls *P* = 0.008, *P* = 0.0168, and *P* = 0.0001, respectively (Figures 2(a), 2(b), and 2(c)).

### 3.4. Correlation of Calgranulin A (S100A8), Calgranulin B (S100A9), and Calprotectin S100A8/S100A9 with BMI, Serum Levels, Histological Grading (NAI), and HOMA-IR

Since calgranulin levels were elevated in the sera of patients with NASH, we tried to find whether there is a correlation with BMI, serum levels, histological grading (NAI), and HOMA-IR. S100A8 showed a significant negative correlation with AST with *P* = 0.034 and positive relation with CRP with *P* = 0.008, similar to S100A9 which showed also a significant positive correlation with CRP with *P* = 0.005; however, no other significant correlation was found to be significant with other parameters. The NASH activity index depicting the histological findings of patients was related positively to the level of the complex S100A8/A9 with a *P* = 0.005 (Table 3).

### 3.5. The Expression and the Protein Concentration of IL-17 and CCL20

**Figure 3:**
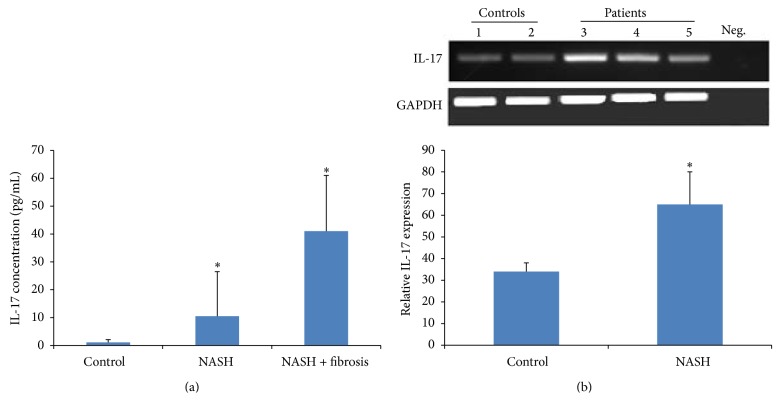
IL-17 expression. (a) IL-17 concentrations in pg/mL in the serum of control subjects and NASH patients. IL-17 was measured by ELISA as described in Section 2. Data represent mean ± SEM in triplicate (ELISA) from 39 different donors/sera of NASH patients. The levels of IL-17 were significantly increased in NASH patients and NASH patients with fibrosis compared with control subjects, with *P* = 0.0332 and *P* = 0.0001, respectively. (b) On the mRNA level, IL-17A mRNA is overexpressed in NASH patients with *P* = 0.038 (^*^ *P* < 0.05).

**Figure 4:**
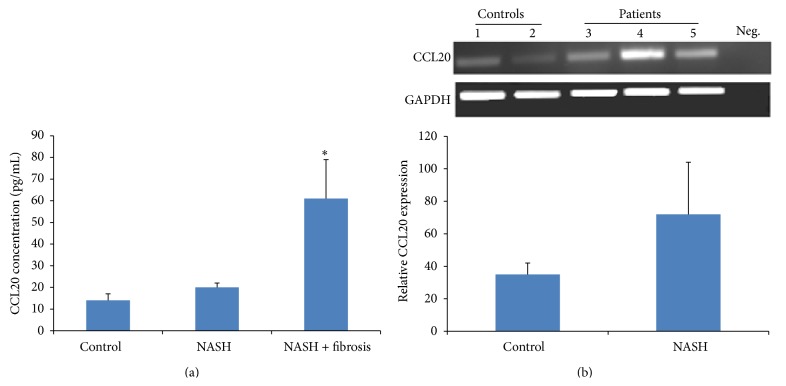
CCL20 expression. (a) CCL20 concentrations in pg/mL in the serum of control subjects and NASH patients. CCL20 was measured by ELISA as described in Section 2. Data represent mean ± SEM in triplicate (ELISA) from 39 different donors/sera of NASH patients. The levels of CCL20 were significantly increased in NASH patients and NASH patients with fibrosis compared with control subjects, with *P* = 0.0332 and *P* = 0.0001, respectively. (b) On the mRNA level, CCL20 mRNA is not significantly increased in NASH patients with *P* = 0.1208 (^*^ *P* < 0.05).

It was shown that IL-17 exacerbates hepatic steatosis and inflammation in nonalcoholic fatty liver disease [5]. In this sense, we tried to investigate the expression of IL-17. The serum level of IL-17 was significantly increased in both the NASH patients and the NASH patients with fibrosis with *P* = 0.0332 and *P* = 0.0001, respectively (Figure 3(a)). This increase was confirmed by an elevated level of IL-17 mRNA (Figure 3(b)). CCL20 showed the same pattern of increase in the serum of patient with fibrotic NASH with *P* = 0.014; however, no increase was detected in NASH patients compared to control patients (Figure 4(a)). The RNA expression of both patients was also increased (Figure 4(b)).

## 4. Discussion

In this study, we were trying to elucidate the factors involved in the progression of NASH to cirrhosis then hepatocellular carcinoma (HCC). We know that many inflammatory cytokines are secreted by hepatocytes [35] and play a role in perpetuating the inflammation and in mediating the apoptosis and fibrosis of the liver cells [36, 37]. There is a stout correlation between chronic inflammation and cancer in a particular organ such as chronic pancreatitis and pancreatic cancer, ulcerative colitis and colon cancer, and hepatitis and liver cancer [38, 39]. Does fibrosis facilitate the occurrence of hepatocellular carcinoma? Few studies have identified that the underlying cause of HCC is steatohepatitis [40, 41] and that telomerase activation is required for hepatocellular carcinoma progression [42]. In our study, we have found that the biopsies of fibrotic NASH patients have a significant increased level of telomerase activity in comparison with NASH patients. To our knowledge, no other studies have shown an increased telomerase activity in NASH fibrotic patients but it was shown to be expressed in cirrhotic liver [43]. The hepatocellular destruction, the chronic inflammation, and fibrosis create an environment leading to carcinogenesis. Many factors are known to exacerbate inflammation and may contribute to liver injury; it has been shown by Salomone et al. that unconjugated bilirubin is associated with the progression of inflammation and fibrosis [44]. Cytokines and chemokines such as IL-17 and CCL20 play a major role in this inflammatory progressive state. Indeed, IL-17 was significantly increased in the sera of NASH patients compared with control and this increase was more pronounced in the serum of fibrotic patients with NASH; this confirms the role of IL-17 in liver diseases [5]. Th 17 was also shown to be increased in hepatocellular carcinoma [45]; this may suggest that IL-17 not only may be a marker of the disease but also could serve as an indicator for progression into hepatocellular carcinoma. Indeed, in our study, the increase in IL-17 in the sera of fibrotic NASH patients was correlated with an increase in the mRNA level of IL-17. Chemokines (e.g., CCL2, CCL3, CCL5, CCL13, CCL18, and CCL20) represent a family of cytokines known to centrally participate in many inflammatory diseases and tumors [46]. The chemokines and their corresponding receptors known as axes have been shown to be involved in the growth and progression of many tumors [47]. Many studies have focused on the role of CCL20-CCR6 in many types of cancer [48] specifically in hepatocellular carcinoma (HCC). It was shown that the level of CCL20-CCR6 is highly expressed in HCC and in tissues with grade III tumors in comparison with grade II tumors suggesting that it not only plays a significant role in the growth and progression of HCC but also is involved in the formation and development of HCC [49] and has a role in identifying primary and secondary liver tumors [50]. In our study, CCL20 was more increased in the sera of NASH patients with fibrosis in comparison with NASH patients without fibrosis which suggests a role for CCL20 in the development of the disease and its progression to fibrosis which might lead the way to HCC.

To confirm our hypothesis that the inflammation seen in fibrotic NASH patients might lead the way to cancer, we investigated the level of the two calgranulins, S100A8 and S100A9, in the serum of patients affected by NASH. S100A8 and S100A9 are known to have proinflammatory functions and are expressed in many diseases like inflammatory arthritis and inflammatory bowel diseases [51, 52] but also they are expressed in several types of cancer [16] such as lung, colorectal, and liver cancer [53–55]. In our study, we found that the level of S100A9 and the heterocomplex S100A8/A9 are significantly unregulated in the sera of NASH patients with more prominent increase in the sera of NASH patients with fibrosis. These findings confirm the results of others where S100A9 was found to be a trigger molecule implicated in the evolution and progression of idiopathic pulmonary fibrosis [56] and where S100A8/A9 was identified as initial proinflammatory molecule needed to stimulate inflammation and cardiac injury [57].

Finally, we think that the increase in the expression of the proinflammatory markers in the patients with fibrotic NASH might be an alarm for the progression towards a cancerous state. It indicates that there is a direct link between the expression of IL-17, CCL20, telomerase, S100A8, and S100A9 in the fibrotic condition and the progression towards cancer. In this case, we should start working towards stopping or preventing this progression.

## Figures and Tables

**Table 1:** Clinical values for NASH patients and healthy volunteers.

Variables	NASH	Control	*P* value
Gender (M/F)	30/9	28/9	
Age (years)	38.6 ± 9.4	41.9 ± 10.9	
AST (IU/L)	88.74 ± 17.189	23.79 ± 7.328	0.0001
ALT (IU/L)	115.75 ± 115.751	23.33 ± 23.333	0.0001
ALP (IU/L)	191.25 ± 44.22	159.00 ± 31.91	0.1343
CRP (mg/dL)	1.04 ± 0.999	0.28 ± 0.123	0.0001
t-cholesterol (mg/dL)	204.90 ± 35.544	191.38 ± 41.831	0.477
t-bilirubin (mg/dL)	0.67 ± 0.274	0.52 ± 0.227	0.215
Glucose (mg/dL)	99.12 ± 9.30	94.30 ± 8.55	0.193
Insulin (U/mL)	17.81 ± 11.454	9.68 ± 9.680	0.0129

Values expressed are means ± SD. AST: aspartate aminotransferase; ALT: alanine aminotransferase; ALP: alkaline phosphatase; t-bilirubin: total bilirubin; CRP: C reactive protein.

**Table 2:** Histological scoring system for nonalcoholic steatohepatitis.

Variable	Score	Description
Steatosis	0	None
	1	Up to 33% of acini, mainly macrovesicular
	2	34%–66% of acini, commonly mixed steatosis
	3	Over 66% of acini (panacinar), commonly mixed steatosis

Hepatocyte ballooning	0	None
	1	Occasional in zone III
	2	Obvious in zone III
	3	Marked, predominantly in zone III
	0	None

Lobular inflammation	1	Scattered neutrophils, occasional mononuclear cells, 1 or 2 foci per 20x objective
	2	Neutrophils associated with ballooned hepatocytes, mild chronic inflammation, 3 or 4 foci per 20x objective
	3	Acute and chronic inflammation, neutrophils concentrating in zone III, over 4 foci per objective
	0	None

Portal inflammation	1	Mild, some portal areas
	2	Mild to moderate, most portal areas
	3	Moderate to severe, most portal areas
	0	No fibrosis

Stage	1	Zone III perivenular, perisinusoidal (pericellular) fibrosis
	2	Stage 1 changes + periportal fibrosis
	3	Bridging fibrosis
	4	Cirrhosis

From Merat et al. [31].

**Table 3:** Statistical relation between variables.

Variables	*P* value	Relation	Formula in case of relation
S100A8 versus AST	0.034	Negative	S100A8 = (0.059 ∗ AST) − 0.808
S100A8 versus CRP	0.008	Positive	S100A8 = (0.891 ∗ CRP) + 3.699
S100A9 versus CRP	0.005	Positive	S100A9 = (0.297 ∗ CRP) + 1.634
S100A8/A9 versus NAI	0.050	Positive	S100A8/A9 = (14.105 ∗ NAI) + 162.74

The statistical correlation between values was performed using univariate linear regression. S100A8 is negatively correlated with AST (S100A8 = (0.059 ∗ AST) − 0.808) and positively correlated with CRP (S100A8 = (0.891 ∗ CRP) + 3.699). S100A9 is positively correlated with CRP (S100A9 = (0.297 ∗ CRP) + 1.634). S100A8/A9 is positively correlated with NAI (S100A8/A9 = (14.105 ∗ NAI) + 162.74). Statistical relation exists if *P* value is less than 0.005 (*α* error = 5%).
